# *Caenorhabditis elegans* as a Screening Model for Probiotics with Properties against Metabolic Syndrome

**DOI:** 10.3390/ijms25021321

**Published:** 2024-01-22

**Authors:** Ignacio Goyache, Deyan Yavorov-Dayliev, Fermín I. Milagro, Paula Aranaz

**Affiliations:** 1 Faculty of Pharmacy and Nutrition, Department of Nutrition, Food Sciences and Physiology, University of Navarra, 31008 Pamplona, Spainparanaz@unav.es (P.A.); 2 Center for Nutrition Research, University of Navarra, 31008 Pamplona, Spain; 3 Genbioma Aplicaciones SL, Polígono Industrial Noain-Esquiroz, Calle S, Nave 4, 31191 Esquíroz, Spain; 4 Navarra Institute for Health Research (IdiSNA), 31008 Pamplona, Spain; 5 Spanish Biomedical Research Centre in Physiopathology of Obesity and Nutrition (CIBERObn), 28029 Madrid, Spain

**Keywords:** gut microbiota, postbiotics, obesity, insulin resistance, diabetes, cardiovascular disease

## Abstract

There is a growing need to develop new approaches to prevent and treat diseases related to metabolic syndromes, including obesity or type 2 diabetes, that focus on the different factors involved in the pathogenesis of these diseases. Due to the role of gut microbiota in the regulation of glucose and insulin homeostasis, probiotics with beneficial properties have emerged as an alternative therapeutic tool to ameliorate metabolic diseases-related disturbances, including fat excess or inflammation. In the last few years, different strains of bacteria, mainly lactic acid bacteria (LAB) and species from the genus *Bifidobacterium*, have emerged as potential probiotics due to their anti-obesogenic and/or anti-diabetic properties. However, in vivo studies are needed to demonstrate the mechanisms involved in these probiotic features. In this context, *Caenorhabditis elegans* has emerged as a very powerful simple in vivo model to study the physiological and molecular effects of probiotics with potential applications regarding the different pathologies of metabolic syndrome. This review aims to summarize the main studies describing anti-obesogenic, anti-diabetic, or anti-inflammatory properties of probiotics using *C. elegans* as an in vivo research model, as well as providing a description of the molecular mechanisms involved in these activities.

## 1. Introduction

During the last few years, different research studies have evidenced the important role that the gut microbiota play in the metabolic health of the host [1,2]. In this context, different research groups have identified specific modifications in the gut bacterial composition that could be associated with a differential risk of developing metabolic syndrome diseases, including obesity or diabetes, and which seem to play a fundamental role in the appearance of metabolic complications associated with these diseases, such as the increase in oxidative stress and chronic low-grade inflammation [3,4,5,6,7,8,9]. For this reason, it is not surprising that the modulation of the intestinal microbiota towards a healthier bacterial composition is currently considered a major factor to bear in mind for the development of therapeutic strategies in the treatment or prevention of metabolic diseases [10,11,12].

One of the best-known strategies for modulating gut microbiota is the use of probiotics. Probiotics are defined by the Food and Agriculture Organization (FAO) and the World Health Organization (WHO) as “live microorganisms which when administered in adequate amounts confer a health benefit on the host” [13]. Thus, different bacterial strains, mainly lactic acid bacteria (LAB) and strains from the genus *Bifidobacterium*, have emerged as potential treatments due to their health-promoting properties, including lipid-reducing activities, or the maintenance of glucose homeostasis, among others [14,15,16,17]. However, the mechanisms of action of some of these probiotic strains are scarcely understood. For this reason, in vivo studies are necessary to understand the molecular mechanisms involved in the metabolic effects observed with the incorporation of these probiotics into the diet. In this sense, the use of simple in vivo models such as *Caenorhabditis elegans* (*C. elegans*) represents a quick and effective advantage to describe these beneficial activities and understand the molecular mechanisms involved [18].

*C. elegans* has been widely employed as an animal model in different diseases and physiological processes, including obesity, diabetes, aging, and neurodegenerative disorders [19,20,21]. This microscopic nematode can be cultured and manipulated at low cost through conventional in vitro methods, which confers a great advantage as an in vivo model, in addition to its transparency, large number of progenies, short life span, and completely sequenced genomes [20,21]. Interestingly, the high conservation in humans of the genes involved in lipid and carbohydrate regulation makes *C. elegans* an excellent model for exploring the energy homeostasis and the regulation of the cellular lipid storage [22]. Thus, despite its simplicity, this nematode is currently considered a powerful experimental model for the study of physiological and molecular effect of health-promoting compounds, including probiotics, with potential applications in the different pathologies of metabolic syndrome [23,24]. 

This review aims to summarize the main studies describing anti-obesogenic, anti-diabetic or anti-inflammatory properties of probiotics using *C. elegans* as an in vivo research model, as well as a description of the molecular mechanisms involved in these activities. The literature search of this review was performed using PubMed, starting with the keywords *Caenorhabditis elegans* and probiotic, which retrieved 164 results. Then, we delimited the search using the following terms: *Caenorhabditis elegans* and probiotic and metabolic syndrome (five results); *Caenorhabditis elegans* and probiotics and obesity (ten results); *Caenorhabditis elegans* and probiotic and diabetes (four results); *Caenorhabditis elegans* and probiotics and insulin (fifteen results); *Caenorhabditis elegans* and probiotics and insulin resistance (seven results); *Caenorhabditis elegans* and probiotics and glucose tolerance (one result); *Caenorhabditis elegans* and probiotics and NAFLD (no results); *Caenorhabditis elegans* and probiotics and cardiovascular (four results); and *Caenorhabditis elegans* and probiotics and inflammation (thirteen results). The overlapping results were eliminated, yielding a final selection of 35 articles, which were carefully analyzed for the review preparation. The results were divided and discussed according to their potential anti-obesity, anti-diabetic, or anti-inflammatory properties in *C. elegans*.

## 2. Probiotics with Lipid-Reducing Activity in *C. elegans*

In the last decade, the use of simple in vivo models such as *C. elegans* has been demonstrated to represent a powerful method to investigate microorganism–host interactions, as well as to evaluate the antioxidant, anti-aging, and life-extending properties of different probiotics strains [25]. Different Bifidobacteria and Lactobacilli strains have been demonstrated to extend nematode lifespan, mainly through modulations in the p-38 mitogen-activated protein kinases (p38-MAPK) signaling pathway [26]. For example, a recent study demonstrated that supplementation with *Bacillus subtilis* DG101, isolated from the traditional Japanese fermented food Nattō, extended worm lifespan by 45% and improved chemotaxis, in comparison with *E. coli* OP50-fed worms [27]. Similarly, supplementation with *Lacticaseibacillus casei* 62 and *Lacticaseibacillus casei* 63 increased nematode lifespan by improving the mitochondrial function, suggesting these bacterial strains as potential probiotics in sarcopenia [28].

However, the information about the potential anti-lipogenic, anti-diabetic, or anti-inflammatory properties of certain probiotic strains have been scarcely described in *C. elegans* [29]. Here, we summarize all the research describing potential anti-obesity or anti-diabetic properties of potential probiotic bacterial strains in *C. elegans*. The fat-reducing activities, cholesterol modulation, and the ability to counteract the effect of high-glucose exposure were some of the properties described for these probiotics.

*C. elegans* stores fat in lipid droplets accumulated in intestinal and skin-like hypodermal cells, mainly in the form of triglycerides [22]. Thus, probiotics might modulate lipid accumulation in *C. elegans* by affecting different signaling pathways, such as suppressing glucose uptake or triglyceride synthesis or activating fatty acid β-oxidation [30]. The main bacterial strains with fat-reducing activities are summarized in Table 1.

### 2.1. Bifidobacterium Strains with Anti-Obesity Properties in C. elegans

**Figure 1:**
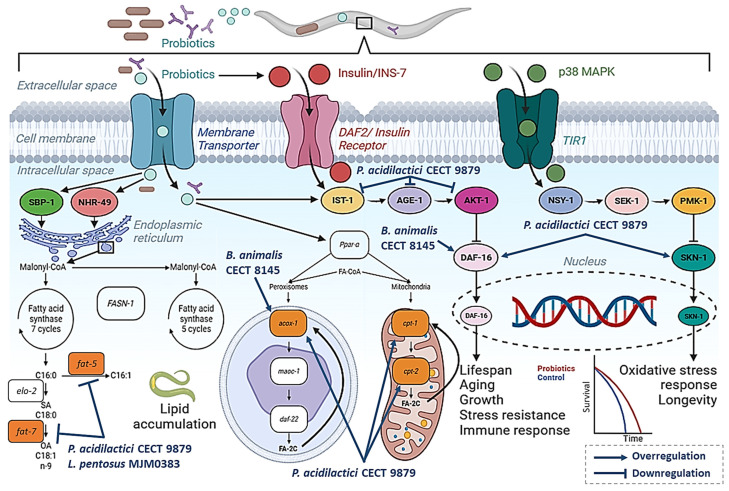
Schematic representation of different signaling pathways affected by the probiotic strains with anti-obesity or anti-diabetic properties in *C. elegans*. The figure includes a representation of the IIS signaling pathway, fatty acid synthesis, fatty acid β-oxidation, and oxidative stress responses.

One of the first studies using *C. elegans* for the study of probiotic properties was published by Martorell et al. [23] (Table 1). In their work, this group performed a screening of the potential lipid-reducing activities of a Biopolis collection of 38 *Lactobacillus* (23) and *Bifidobacterium* (15) strains, previously isolated from the feces of healthy breast-fed babies. They found that the supplementation of the nematode growth medium (NGM) agar with a specific strain of *Bifidobacterium animalis* subsp. *lactis* CECT 8145 induced a reduction of approximately 33% in the lipid content of the worm, in comparison with nematodes fed *E. coli OP50* as a standard diet. This reduction in fat accumulation was also maintained in soy-fermented milk treated with *Bifidobacterium animalis* subsp. *lactis* CECT 8145 [31]. Moreover, treatment with this *Bifidobacterium* strain also induced an improvement of the nematode oxidative stress-response, and an increase in lifespan by 64%. Gene expression analyses demonstrated the involvement of genes involved in energy metabolism, including the beta-oxidation genes *acox-1* and *daf-22*, the energy modulator *daf-16*, and the unsaturated fatty acid synthesis gene *fat-7*, which was overexpressed in CECT 8145-treated worms (Figure 1).

Interestingly, the use of an inactivated form of *Bifidobacterium animalis* subsp. *lactis* CECT 8145 (BPL1), obtained via heat treatment (70 °C for 18 h), induced a similar reduction in the nematode fat content to that of the active form, supporting the idea that the strain efficacy still remained stable in non-viable cells, and that cell wall components might be responsible for the anti-obesogenic properties of this strain [23]. In this context, a subsequent study by this group demonstrated that an infant milk formula supplemented with heat-treated *Bifidobacterium animalis* subsp. *lactis* CECT 8145 (HT-BPL1, as a postbiotic) significantly reduced the fat content in *C. elegans*, confirming the anti-obesogenic properties of this strain in the nematode [31]. Further investigations demonstrated that lipoteichoic acid (LTA), a postbiotic isolated from the BPL1, was responsible for its lipid-reducing activities, both in NGM plates and glucose-loaded conditions [32]. In their work, Balaguer et al. demonstrated that the anti-lipogenic activities of both BPL1 and LTA were independent of SKN-1/p-38 MAPK pathway but were mediated by the modulation of the insulin-like signaling pathway (IGF-1), due to the lack of fat-reducing activity in *daf-2* and *daf-16* mutants [32].

### 2.2. Pediococcus Acidilactici Strains with Anti-Obesity Properties in C. elegans

One of the bacterial species that has emerged in recent years as a potential probiotic with anti-obesogenic and anti-diabetic properties is *Pediococcus acidilactici* [34,37,38,39,40]. Thus, different research groups have evaluated the potential beneficial activities of *Pediococcus acidilactici* strains on the lipid and carbohydrate metabolism using *C. elegans* (Table 1) [33,34,35]. In their study, Daliri et al. isolated lactic acid bacteria (LAB) strains from Korean fermented soybean paste, which included *Pediococcus acidilactici* SDL1402 and *P. acidilactici* SDL1406, together with *Weisella cibaria* SCCB2306 and *Lactobacillus rhamnosus* JDFM6 strains [35]. These four strains were able to reduce cholesterol levels in *C. elegans*, and increase the lifespan of the worms in comparison to *E. coli OP50*-fed worms [35].

In a similar study performed by Barathikannan et al. [33], a novel strain of *Pediococcus acidilactici* MNL5 was isolated through the screening of thirty-two LABs from fermented Indian herbal medicine with health-promoting activities in *C. elegans*. *P. acidilactici* MNL5 was able to counteract the lifespan-reduction induced by glucose supplementation in the medium, together with a reduction in the fat accumulation, in comparison with *E. coli OP50*-fed worms [33]. Gene expression analysis suggested that *P. acidilactici* MNL5 inhibited de novo lipogenesis by down-regulating the fatty acid desaturase-coding genes *fat-4*, *fat-5*, and *fat-6*, inducing a reduction in lipid accumulation (Figure 1) [33].

Similarly, our group demonstrated the anti-obesogenic and anti-diabetic properties of the strain *Pediococcus acidilactici* CECT 9879 (p1Ac^®^) in *C. elegans* [24,34] and rodents [34,40]. This strain was able to counteract the effect of glucose on *C. elegans* fat accumulation, lifespan, oxidative stress, and aging [24]. Thus, supplementation with *P. acidilactici* CECT 9879 at a dose of 5 × 10^6^ CFU/mL (in combination with the nematode standard diet *E. coli OP50*) was able to significantly reduce the nematode fat content in comparison with nematodes grown with OP50 only. Moreover, the probiotic was able to counteract the effect of high-glucose conditions by ameliorating aging (reducing lipofuscin pigment), enhancing the stress–oxidative response and prolonging lifespan without affecting worm development [24].

Gene expression analysis and mutant assays demonstrated that *P. acidilactici* CECT 9879 exerted health-promoting activities by affecting the IIS signaling pathway, by increasing the expression of *daf-16*, but also affecting the SKN-1/Nrf-2 signaling pathway. Moreover, the anti-obesity and anti-diabetic properties of *P. acidilactici* CECT 9879 included the inhibition of fatty acid biosynthesis (via the downregulation of *fasn-1*, *fat-5*, *fat-7*, and *mdt-15* genes), and inducing FA degradation (by increasing the expression β-oxidation genes *acox-1*, *daf-22*, *maoc-1*, and *cpt-2*) (Figure 1) [24]. The properties of this probiotic strain and the molecular mechanism of action were confirmed and maintained in a subsequent study where *P. acidilactici* CECT 9879 was combined with the prebiotic ingredients chromium picolinate (0.5 μg/mL) and oat-beta glucans (50 μg/mL) [34]. Taken together, the research performed in *C. elegans* demonstrates the potential anti-obesity and anti-diabetic properties of *P. acidilactici* strains and supports the need for additional studies in higher models to determine its possible application in humans.

### 2.3. Other Lactic Acid Bacteria with Anti-Obesity Properties in C. elegans

Lactic acid bacteria (LAB) are important compositors of gut microbiota due to their beneficial properties by inhibiting the growth of pathogenic bacteria, enhancing intestinal function, modulating metabolic functions, and regulating the immune system. Thus, like *Pediococcus*, other LAB have been described to exert fat-reducing activities in *C. elegans*. In their study, Marquez et al. [30] described the anti-obesity properties of a probiotic mixture *Lactobacillus delbrueckii subsp*. *indicus* CRL1447, to which a mix of probiotics consisting of *Limosilactobacillus fermentum* CRL1446, *Lactiplantibacillus paraplantarum* CRL1449, and CRL1472 strains was then added in different formulations (Table 1). This combination was able to reduce worm TG content, when combined with *E. coli OP50* at a ratio 25:75, in comparison with 100% of *E. coli OP50* [30].

An interesting study performed by Gu et al. [36] described the efficacy of a probiotic strain of *Lactobacillus pentosus* MJM60383 in an obese model of *C. elegans* (Table 1). In this work, they established a new obesity model by feeding this nematode with a culture of *Enterobacter cloacae*, a proposed pathogenic bacterium that induces obesity in germ-free mice, in combination with high-glucose (100 mM) conditions (HGD-E) [36]. The proposed *C. elegans* obese model was characterized by an increase in the lipogenic and a decrease in the β-oxidation genes. With this model, they demonstrated that supplementation with *L. pentosus* MJM60383 strain was able to significantly reduce *C. elegans* fat accumulation, counteracting the effect of the obesity-related pathogenic bacteria *E. cloacae* [36].

## 3. Probiotics Counteracting the Effect of High Glucose Exposure *in C. elegans*

In *C. elegans*, the insulin-like signaling pathway (IGF-1) regulates aging, immunity, and lipid metabolism [41]. This signaling pathway is conserved across nematodes and mammals, including humans, and has been a key area of research in obesity. This signaling pathway is mainly controlled by DAF-2, homologous to the human insulin receptor, and DAF-16, which represents the *Forkhead* family of transcription factors (FOXO in humans) that play a central role in mediating the molecular mechanisms of this pathway [41,42]. The IIS pathway, with the *daf-2*/*daf-16* axis as main actors, is involved in glucose transport and insulin sensitivity, being considered a target signaling pathway for the study of insulin resistance and type-2 diabetes [32]. Thus, *C. elegans* has been demonstrated to be a reliable model for evaluating the effect of high glucose exposure, which mimics overfeeding and diabetic conditions and is known to shorten lifespan, increase reactive oxygen species (ROS), and reduce stress response by modulating the insulin/IGF-1 signaling (IIS) pathway [32]. Importantly, since glucose is a known precursor of TGs synthesis, exposure to hyperglycemic conditions (glucose from 10 to 100 mM) has been demonstrated to increase the fat content and enlarge the body size of the worm [43].

Different probiotics have been demonstrated to be able to counteract the exposure to high doses of glucose. As previously mentioned, *L. pentosus* MJM60383 was able to ameliorate the obese phenotype induced by *E. cloacae* supplemented with high dose of glucose. Gene expression analyses demonstrated the downregulation of lipid-accumulation-related genes *fat-6* and *fat-7*, which encode for a stearoyl-CoA desaturase (SCD) known to modulate the relative abundance of saturated and monounsaturated fatty acids in *C. elegans* [44]. Moreover, the fat-reducing activity of this probiotic strain was also mediated by the upregulation of the β-oxidation genes *nhr-49* and *acs-2* [36]. The gene *nhr-49* encodes a transcription factor involved in the control of pathways regulating fat consumption and maintenance of the normal balance of fatty acid saturation by modulating the expression of genes involved in fatty acid beta-oxidation. Thus, the deletion of *nhr-49* increases the fat accumulation in *C. elegans*. *Acs-2*, one of the downstream genes of *nhr-49*, is an acyl-coA synthetase catalyzing the conversion of fatty acid to acyl-CoA, resulting in a reduction in fatty acids [35]. A similar mechanism involving *C. elegans nhr-49*/*acs-2* β-oxidation genes had also been reported to explain the fat-reducing activity of pasteurized *Akkermansia muciniphila* (p-AKK), the most-well described postbiotic for in vivo energy metabolism regulation [45].

Interestingly, *P. acidilactici* CECT 9897 was able to reduce the *C. elegans* fat content in NGM and glucose–NMG medium [24]. In this work, our group demonstrated that the anti-obesity properties of this strain were mediated by the IIS signaling pathway but also by inducing the peroxisomal and mitochondrial β-oxidation of fatty acids. In fact, the exposure of *C. elegans* to *P. acidilactici* CECT 9897 in high-glucose conditions demonstrated that this probiotic was able to revert the nuclear translocation of DAF-16 induced by the glucose and return to the cytosol. Indeed, the anti-obesity and anti-diabetic properties of *P. acidilactici* were accompanied by a reduction in the nematode oxidative stress, aging, and lifespan extension, demonstrating its ability to regulate the IIS signaling pathway [24].

## 4. Probiotics with Anti-Inflammatory Properties in *C. elegans*

As previously mentioned, metabolic diseases are characterized by an increase in cellular oxidative stress, aging, and chronic, low-grade inflammation [46]. Inflammation can be defined as an immune response during an infection or injury which aims to maintain an organism’s homeostasis [47]. In obesity, the increasing inflammation is considered a key risk factor for developing other metabolic diseases, including atherosclerosis or type-2 diabetes [46]. In this regard, *C. elegans* has also been used to investigate the potential anti-inflammatory effects of different probiotics, mainly attributed to their antioxidant, anti-aging, and life-extending properties or to their ability to enhance the functionality of the nematode immune response by conferring resistance to the infection of bacterial pathogens. At molecular level, the inflammatory response to harmful stimuli in *C. elegans* is mainly mediated by the IIS pathway but also by the p38 mitogen-activated protein kinase (p38 MAPK) [48].

The p38/MAPK pathway is a well-conserved inflammatory response in humans and *C. elegans.* It has been shown to be required in the resistance mechanism of *C. elegans* against several infections such as *Cutibacterium acnes*, *Proteus* spp. or *Pseudomonas aeruginosa* among others [48,49,50,51]. In this regard, metformin, the first-line oral drug for type-2 diabetes, has been shown to enhance *C. elegans* tolerance to pathogen infection by acting through *pmk-1*, the main actor of the nematode p38/MAPK signaling pathway [52].

Focusing on our main concern, probiotics, and their beneficial effects on *C. elegans*, there may be a link with their modulation of metabolic pathways and the requirement for the mentioned intestinal activity. Research conducted by Hu et al., where they described the fat-reducing properties of the *P. acidilactici* strain, also included a mechanistical exploration on the life-extending and enhanced oxidative stress response effects on *C. elegans* [53]. Taking advantage of mutant strains, they were able to characterize the molecular mechanism, showing that it is dependent on *daf-16* but also on JNK/MAPK signaling. Treated mutants lacking the expression of *jnk-1* had equal lifespan to the control worms.

Similarly to these findings, Kwon et al. described the probiotic effects of *Propionibacterium freudenreichii* KCTC 1063 on *C. elegans* [54]. After conducting similar lifespan assays with mutants, they showed that the life-extension effect was not observed in *pmk-1*, *sek-1*, *mek-1*, *dbl-1*, *daf-12*, or *daf-2* mutants, suggesting crucial roles for these genes. Interestingly, the upregulation of the expression levels of *daf-12* measured via qPCR also corroborated the role of this nuclear factor which induces the production of antimicrobial peptides and is related to the p38/MAPK pathway.

Similarly, Park et al. tested the effects of *Lactobacillus fermentum* strain JDFM216 on *C. elegans* [55]. After assessing its life-extending effect on the nematode’s life, they also showed how it can confer resistance against *S. aureus* and *E. coli* O157:H7 while colonizing the gut. The mechanism appeared to be dependent on *pmk-1* phosphorylation, which was heavily upregulated in treated worms. Furthermore, no beneficial effects were observed in treated worms lacking the expression of *pmk-1.*

To sum up, the ability of other probiotics to upregulate the expression of *pmk-1*, like the *Lactobacillus fermentum* strain JDFM216 and *Bacillus amyloliquefaciens SCGB1*, or the expression of *skn-1* (*C. elegans* ortholog of *Nrf2*), like the *Lactococcus cremoris* sp. nov. strain FC or *Lactobacillus gasseri SBT2055*, and extend the nematodes lifespan or enhance the host defenses [55,56,57,58], serves to highlight the beneficial effects of this pathway exerted by some probiotic strains.

Besides the activation of the IIS or MAPKS pathways, other mechanisms are able to promote inflammatory states, as summarized in Table 2. The prolonged inflammation of the gut has been associated with what is called “leaky gut”, a disruption of the intestinal barrier, and this is highly connected with the development and/or progression of several metabolic and autoimmune systemic diseases [59]. Therefore, the functional enhancement of this barrier by probiotics is one of the additional mechanisms against inflammation. The use of *C. elegans* to study the intestinal barrier and its functionality has allowed researchers like Shokouh Ahmadi and colleagues to unveil functional treatment-enhancements for five *Lactobacillus* strains (*L. paracasei* D3-5, *L. rhamnosus* D4-4, *L. plantarum* D6-2, *L. rhamnosus* D7-5, and *L. plantarum* D13-4) and five *Enterococcus* strains (*E. raffinosus* D24-1, *E*. INBio D24-2, *E. avium* D25-1, *E. avium* D25-2, and *E. avium* D26-1) isolated from healthy infant guts. After conducting several in vitro and in vivo assays in mice, the authors related the probiotics´ bile salt hydrolase activity to an increased taurine abundance in the gut that stimulated tight junctions though the upregulation of *Zo1* and suppressed gut leakiness. With this data, the researchers conducted in vivo assays on wild type N2 *C. elegans* supplemented with taurine, which reduced intestinal permeability and in turn increased life span, reduced adiposity, and enhanced physical function [60].

The reduction in gut leakiness was assessed thought the “smurf assay” which, briefly, is carried out by feeding *C. elegans* with a dye and observing it though a fluorescent microscope if it passes though the intestinal epithelial barrier. This assay, together with similar ones based on the same principles [61], has also permitted Le et al. to characterize the response of *C. elegans* to different bacteria, including known pathogens and others with probiotic properties like *Enterococcus faecalis*, which did not affect the gut permeability when compared to pathogens.

To sum up, the inflammatory response in *C. elegans* has been mechanistically described over the past few years, giving researchers in the field of probiotics a powerful tool to further characterize the effects of beneficial bacteria and their mechanisms of action of immune and inflammation responses.

## 5. Study Limitations

The studies collected in Table 1 report very interesting and valuable information about the fat-reducing and glucose-counteracting effects of specific bacterial strains, including *Bifidobacterium*, *Lactobacillus*, and *Pediococcus* genera, among others. Moreover, as described in Table 2, different probiotics have been demonstrated to extend *C. elegans* lifespan counteracting inflammation. Nevertheless, some of the results are difficult to compare between studies due to the different methodologies used or the lack of information about the doses of probiotics used in some of the studies, which makes it difficult to compare the probiotics tested.

It is worth noting that many studies fail to evaluate possible modifications in the growth and/or development of the worms, including offspring, after exposure to the probiotic. These factors are particularly relevant when comparing the fat-reducing activity of a specific probiotic strain in comparison with a nematode’s standard diet of *E. coli* OP50. Bacterial dietary fatty acids are transformed into triglycerides, which are the main forms of fat deposited in the epithelial and intestine of *C. elegans* [33]. Thus, differences in the fatty acid composition of these bacteria might significantly affect the lipid content of the nematode. The *E. coli* OP50 strain is widely used as a standard diet for the proper growth and development of nematodes, with a specific development time [62].

However, the evaluated probiotic strains might have different fatty acid compositions, which could represent a lower energy source and might affect the nutritional status of the worm and, thus, its development [63]. In this case, the reported lipid-reducing effect would not be attributed to anti-lipogenic or lipolytic activity but rather to lower energy consumption. *C. elegans* has been widely used as a model to investigate the effects of calorie restriction, which promotes the extension of nematode lifespan, accompanied by an improvement in stress response and a reduction in the *C. elegans* fat accumulation mediated by the IIS pathway [64]. For this reason, the replacement of the standard food source by new bacterial strains should be investigated with caution, especially when attributing specific metabolic properties to the probiotics. For example, Zanni et al. [16] demonstrated that *C. elegans* supplementation with a foodborne LAB consortium, including *Lactobacillus delbrueckii*, *Lactobacillus fermentum*, and *Leuconostoc lactis*, induced a significant increase in the fat accumulation of the worm, in comparison with *E. coli OP50*-fed worms, through the downregulation of *daf-16*. In this work, the substitution of the nematode standard diet with this foodborne microbiota induced a reduction in worm survival accompanied by a reduction in the progeny number in comparison with nematodes grown with *E. coli* OP50. In this context, a recent study demonstrated that supplementation with gut microbiota (MCB) from murine feces represented lower energy density (glucose and triglycerides) in comparison with *E. coli* OP50. Consequently, MCB-fed worms exhibited smaller body length and size, lower fertility, lower fat content, and an extended lifespan in comparison to those fed with the standard diet [65]. Moreover, Gu et al. demonstrated the differential fatty acid composition of *C. elegans* exposed to *E. cloacae* in comparison with *E. coli* OP50 [36].

In contrast, *Pediococcus acidilactici* was shown to be able to reduce the nematode lipid content when administered in combination with the *E. coli* OP50, in comparison with the *E. coli* alone, without affecting worm development. A similar finding was identified by Balaguer and colleagues, who demonstrated that LTA from BPL1, in combination with OP50, was able to reduce *C. elegans* fat accumulation in comparison with OP50 alone [32].

## 6. Conclusions

The supplementation of the diet with beneficial bacteria has been proposed as an emerging therapy to combat or prevent the development of metabolic syndrome-related diseases, including obesity and type 2 diabetes. In vivo models are needed to characterize the strain-specific beneficial effects of these probiotics and to determine the molecular mechanisms involved in these effects. In this work, it has been shown that the supplementation of the nematode *C. elegans* with different probiotics, including species of the *Bifidobacterium*, *Lactobacillus*, or *Pediococcus* genera, can reduce lipid content compared to the standard diet through the modulation of the synthesis of triglycerides and the peroxisomal and mitochondrial β-oxidation of fatty acids. Furthermore, some of the described bacterial species are capable of counteracting the effects of exposure to high doses of glucose by reducing not only fat accumulation but also by reducing oxidative stress and aging and increasing the life expectancy of the nematodes. In most cases, the anti-obesity and anti-diabetic properties of these probiotics are mediated by the modulation of the insulin-like signaling pathway (IGF-1). Moreover, some specific strains have been shown to control the inflammatory response in *C. elegans* through the modulation of the p38/MAPK signaling pathway. Our review demonstrates that *C. elegans* represents a reliable model to evaluate the potential anti-obesity, anti-diabetic, or anti-inflammatory properties of specific bacterial strains proposed as probiotics for the prevention of metabolic syndrome.

## Figures and Tables

**Table 1:** Summary of the studies using *C. elegans* for the screening of probiotic strains with anti-obesity or anti-diabetic properties.

Probiotic Strain	Food Sources and Culture Conditions	Main Findings	Mechanisms (Signaling Pathways Involved)	Reference
*Bifidobacterium animalis* subsp. *lactis* CECT 8145				
Active form of *Bifidobacterium animalis* subsp. *lactis* CECT 8145 (probiotic)	*E. coli* OP50 strain or *B. animalis* subsp. *lactis* CECT 8145; No dose specification 20 °C; Three days until young adults.	↓ Fat content (Nile red and TG quantification) ↑ Resistance to acute oxidative stress ↑ worm survival	Downregulation of positive regulators of growth rate and the xenobiotic metabolism. Up-regulation of metabolic pathways for energy production. ↑ Lipid glycosylation ↑ *acox-1*	[23]
Heat-treated *Bifidobacterium animalis* subsp. *lactis* CECT 8145	NGM surface previously seeded with *E. coli OP50*; Worms were incubated for 3 days at 20 °C.	↓ Fat content (Nile red and TG quantification) ↑ SCFAs production: acetate, lactic acids	NF-κB	[31]
Lipoteichoic acid from *Bifidobacterium animalis* subsp. *lactis BPL1* and LTA metabolite	*Escherichia coli OP50* strain NGM and glucose-NGM medium; *B. animalis* and HI-*B. animalis* (10^8^ cells/plate) were added to the NGM surface. Lipoteichoic acid (LTA) as bioactive compound (50 to 0.1 µg mL^−1^).	↓ Fat accumulation by probiotic and LTA, also in NGM+ glucose	No effect on fat reduction on *daf-2 or daf-16* mutants. Not dependent on *skn-1* shown in mutants	[32]
*Pediococcus acidilactici*				
*Pediococcus acidilactici MNL5*	*E. coli OP50* or *P. acidilactici MNL5*.	↑ nematode lifespan and median survival ↓ Fat content vs. NGM OP50 + glucose (Nile red and oil red)	↓ *fat-4 fat-5* and *fat-6* Glucose upregulated de novo fatty acid synthesis	[33]
*Pediococcus acidilactici* CECT 9879 (p1Ac)	Worms were grown from L1 to L4 at 20 °C; Probiotic dose: 5 × 10^6^ CFU/mL; NGM and high-glucose NGM (10 mM) previously seeded with *E. coli OP50* as normal nematode diet.	↓ Fat content (Nile red and oil red) Normal worm development ↓ oxidative stress (ROS) ↓ aging (lipofuscin) ↑ nematode lifespan and median survival	IIS signaling pathway: pA1c inhibits the high-glucose-induced nuclear translocation of *daf-16* ↓ *fasn-1*, *fat-5*, *fat-7*, and *mdt-15* gene expression ↑ *acox-1*, *daf-22*, *maoc-1*, and *cpt-2* gene expression ↑ *skn-1* and *nhr-49* gene expression	[24]
*Pediococcus acidilactici* CECT 9879 (p1Ac) combined with prebiotics	Worms were grown from L1 to L4 at 20 °C; Probiotic dose: 5 × 10^6^ CFU/mL; 0.5 µg/mL of PC; 50 µg/mL of BGC NGM and high-glucose NGM (10 mM) previously seeded with *E. coli OP50* as normal nematode diet.	↓ Fat content (Nile red and oil red) Normal worm development ↓ oxidative stress (ROS) ↓ aging (lipofuscin) ↑ nematode lifespan and median survival	pA1c inhibits the high-glucose-induced nuclear translocation of *daf-16* ↓ expression of fatty acid biosynthesis genes: *fat-5* ↑ expression of β-oxidation genes: *acox-1* and *cpt-2*	[34]
*Other Lactic Acid Bacteria (LAB)*				
*LAB strains from Korean Fermented Soya Beans:* *Pediococcus acidilatici SDL1402* *P. acidilactici SDL1406* *Weisella cibaria SCCB2306* *Lactobacillus rhamnosus JDFM6*	*E. coli OP50* or 50 µL of LAB; (8 Log CFU/mL).	↓ Cholesterol accumulation irrespective to the order of treatment ↑ worm survival		[35]
*Lactobacillus delbrueckii* subsp. *indicus* CRL1447 combined with mixes of *Limosilactobacillus fermentum* CRL1446, *Lactiplantibacillus paraplantarum* CRL1449, and CRL1472 strains	*E. coli OP50* (control group) or a combination of *E. coli OP50* and each *lactobacilli* strain in a ratio of 25:75; 20 °C; L1 to L4/adult.	↓ TG content		[30]
*Lactobacillus pentosus* MJM60383	*E. coli OP50* or *E. cloacae* 20 °C; Synchronized L1 worms were fed with OP50, or *E. cloacae*; NGM plate supplemented with 100 mM glucose.	↓ Fat content (Nile red and oil content) ↓ ratio of C18:1∆9/C18:0	↑ *acs-2* and *nhr-49* genes, enhancing fatty acid β-oxidation ↓ *fat6* and *fat7* and *tub1*	[36]

↑: increased; ↓: reduced.

**Table 2:** Summary of the studies using *C. elegans* for the screening of probiotic strains with anti-inflammatory properties.

Probiotic Strain	Food Source and Culture Conditions	Main Findings	Mechanisms (Signaling Pathways Involved)	Reference
Probiotic cocktail containing five *Lactobacillus* and five *Enterococcus* strains isolated from healthy infants	*E. coli* OP50 with or without taurine; Supplementation of synchronized worms from L1 stage; (proof of concept of the probiotic bile hydrolase activity).	↓ leaky gut (smurf assay) ↑ motility ↑ worm survival	Not described in *C. elegans*.	[60]
*Lactobacillus gasseri* SBT2055	*E. coli* OP50 or *Lactobacillus gasseri* SBT2055 (live or UV killed); 20 °C; L1 to L4/adult.	↑ worm survival ↓ aging (lipofuscin) ↑ Oxidative stress response (Paraquat asay) ↑ Mitochondrial function measured by MitoTracker^®^ CMXRos and cyanine dye JC-1	*Skn-1*, *nsy-1*, *sek-1*, and *pmk-1* dependant mechanism for life-extension via p38 MAPK pathway signaling. Independent effects from *daf-2* or *daf-16*. Upregulation of oxidative stress related genes: *skn-1*, *gst-4*, *sod-1*, *trx-1* (thioredoxin), *clk-1* (mitochondrial polypeptide), *hsp16.2* (heat-shock protein), *hsp-70*, and *gcs-1* (an ortholog of γ-glutamyl-cysteine synthetase).	[58]
*Propionibacterium freudenreichii* KCTC 1063	*E. coli* OP50 or *Propionibacterium freudenreichii* KCTC 1063; 25 °C; Assays performed on L4 adults.	↑ worm survival ↓ aging (lipofuscin) resistance to *Salmonella typhimurium*	*Skn-1* mutants failed to benefit from extended life. Upregulation of p38/MAPKK pathway genes *daf-2*, *pmk-1*, *sek-1*, *mek-1*, *dbl-1*, *daf-7*, *sma-3*, and *daf-12*. Upregulation of antimicrobial peptide-related genes *lys-7* and *lys-8*.	[54]
*Lactobacillus fermentum* Strain JDFM216	*E. coli* OP50 or *Lactobacillus fermentum* JDFM216; 25 °C; L1 to L4/adult.	↑ worm survival ↑ Resistance to food-borne pathogens, including *Staphylococcus aureus* and *E. coli* O157:H7	Upregulation of the NHR and PMK-1 pathway.	[55]
*Bacillus amyloliquefaciens* SCGB1	Exposure to *E. coli* O157:H7 or *Bacillus amyloliquefaciens* SCGB1.	↑ worm survival upon exposure to pathogen *E. coli* O157:H7.	Upregulation of *pmk-1*.	[56]
*Lactococcus cremoris* subsp. *cremoris*	*E. coli* OP50 or *Lactococcus cremoris* subsp. *Cremoris*; 25 °C; Young adult worms.	↑ Resistance to *Salmonella enterica* subsp. *enterica* serovar Enteritidis or *Staphylococcus aureus* ↓ aging (lipofuscin)	No beneficial effects on *skn-1* lacking mutants. Upregulation of heme oxygenase-1 *ho-1*, effector of the SKN-1/Nrf2 pathway.	[57]

↑: increased; ↓: reduced.

## Data Availability

Data sharing is not applicable.
